# Management of Medication-Related Osteonecrosis of the Jaws With Hyperbaric Oxygen Therapy: A Case Report

**DOI:** 10.7759/cureus.70940

**Published:** 2024-10-06

**Authors:** Rakshak Anand, Yajas Kumar, Nitin Bhagat, Kapila Chakarvarty, Yashmi Jaiswal

**Affiliations:** 1 Department of Oral and Maxillofacial Surgery, Manav Rachna Dental College, Faridabad, IND; 2 Oral and Maxillofacial Surgery, Private Practice, New Delhi, IND

**Keywords:** bisphosphonate-related osteonecrosis of the jaw, bisphosphonate therapy, bronj, hyberbaric oxygen therapy, nonhealing wound

## Abstract

Medication-related osteonecrosis of the jaw (MRONJ) refers to the condition where the maxillary or mandibular bone becomes exposed and necrotic as a result of bisphosphonate therapy. The number of patients on bisphosphonates is increasing and so are the MRONJ cases. Since the initial data were published in the early 2000s, research into MRONJ has expanded significantly to enhance the understanding of this emerging condition. Various treatment options are available, but none have been established as the definitive "gold standard" for managing this disease. Hyperbaric oxygen therapy (HOT) is one of the treatment modalities for MRONJ, which has generated successful outcomes and an improvement in the quality of life along with a reduction in morbidity, as mentioned in the literature. We validate this by reporting a case of MRONJ successfully treated with HOT.

## Introduction

Medication-related osteonecrosis of the jaw (MRONJ) describes a disease where the necrotic alveolar bone becomes exposed in individuals who have undergone prolonged bisphosphonate (BP) therapy. Bisphosphonates are frequently prescribed for osteoporosis, Paget's disease, and bone metastases because they effectively inhibit bone resorption. However, prolonged use of these drugs can lead to impaired bone remodeling and healing, particularly in the jaw, where high bone turnover occurs. MRONJ typically develops without any prior history of radiation therapy to the oral or maxillofacial region, distinguishing it from osteoradionecrosis. The condition often arises after dental procedures, such as extractions, or even spontaneously and presents with symptoms like pain, infection, and exposed bone in the oral cavity. Managing MRONJ is often complex and demands a multidisciplinary approach that incorporates conservative treatments, antibiotic therapy, and surgical interventions in more advanced cases. Treatment options for MRONJ vary from non-invasive methods, like oral care and medication management, to surgical procedures, like debridement and surgical resection, depending on the graveness of the condition. The aim is to manage symptoms, prevent additional bone necrosis, and enhance the patient’s quality of life. The use of hyperbaric oxygen (HBO) in the management of MRONJ has been supported by a growing body of evidence, including case series and retrospective studies, which have demonstrated improved outcomes, including reduced pain, enhanced wound healing, and decreased need for surgical interventions [1,2]. We report a case of extensive MRONJ as a result of using long-term oral bisphosphonates treated primarily using HBO therapy with a successful outcome.

## Case presentation

**Figure 1:**
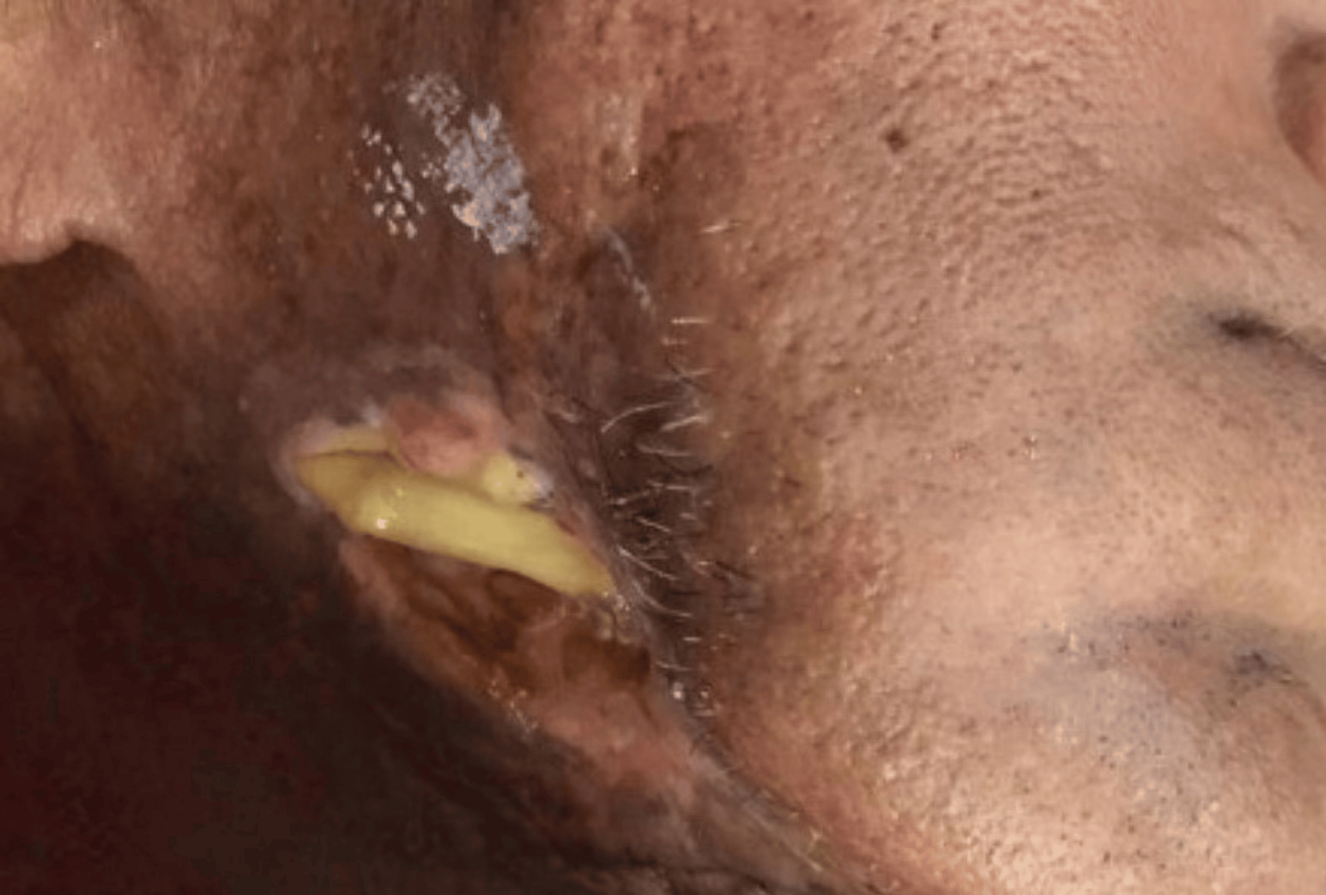
Non-healing open wound present with respect to the right angle region.

A 62-year-old male patient reported to the outpatient department of Manav Rachna Dental College, Faridabad, with a chief complaint of an open wound on the right side of the face for seven months. The patient gave a medical history of osteoporosis dating eight years ago, for which he was prescribed alendronate. He had a habit of tobacco chewing and smoking for 20 years. He also gave a history of extraction with respect to 44, 45, 46, and 47 a year and a half before the time of presentation, which did not heal, and the patient experienced intermittent foul smell with pus discharge. Owing to this, the patient was put on amoxicillin, metronidazole, and ciprofloxacin multiple times by a local practitioner with no improvement. On examination, the exposed bone was seen with respect to the right angle region extraorally with purulent discharge, and no signs of paresthesia were present along the right inferior alveolar nerve (Figure 1).

**Figure 2:**
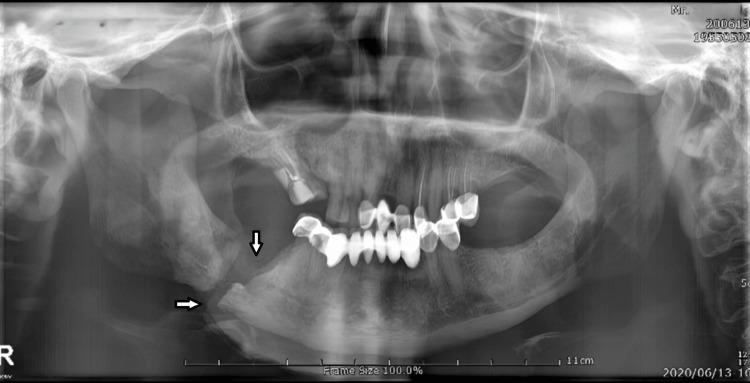
Panoramic radiograph further showing areas of osteolytic activity and radiolucencies with respect to the right angle of the mandible and a breach in continuity depicting the right angle fracture. The panoramic image shows a pathological fracture along with an evident periosteal new bone formation. Trabecular bone in this region has a mixed structure in which osteolytic and osteosclerotic areas can be observed together. The anterior part of the inferior alveolar canal (anterior to the fracture) seems to be narrowed. There is degradation and severe porosity in the endosteal margin of the mandibular cortical bone.

A panoramic radiograph further revealed a breach in the right mandibular angle region with a radiolucent area with a breach in the lower border measuring about 1.5 by 1.0 cm (Figure 2).

**Figure 3:**
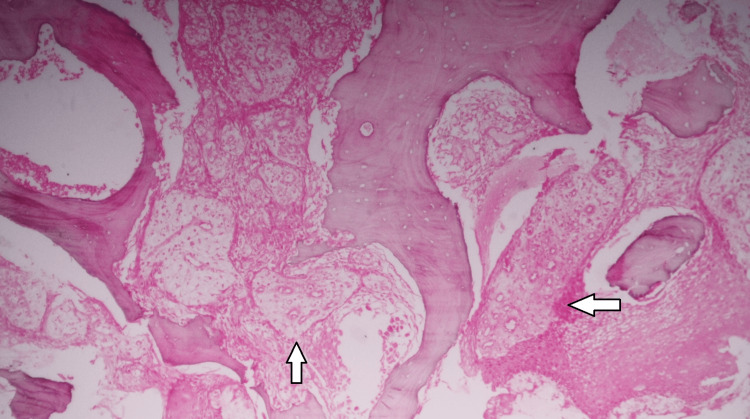
Microscopic presentation showing necrotic bony trabeculae with empty osteocyte lacunae. Osteoclasts containing numerous intracytoplasmic vacuoles are seen at the periphery and in the intertrabecular spaces of the bony trabeculae.

**Figure 4:**
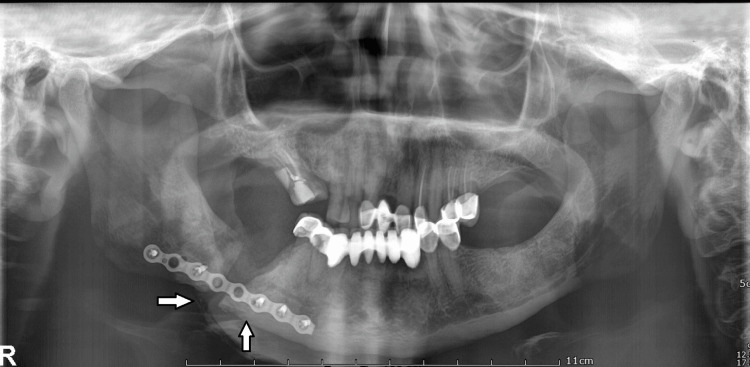
Panoramic radiograph showing fractured segments secured with an eight-hole titanium plate and screws.

The case was classified as American Association of Oral and Maxillofacial Surgeons (AAOMS) stage 3 [2] and Marx stage 2B [3]. The collected pus was sent for culture and sensitivity, which came back as sterile. The superficial necrotic tissue was sent for histopathological examination, which diagnosed it to be an inflammatory necrotic bone (Figure 3), and the bisphosphonates were stopped during the treatment phase. Intraoperative intermaxillary fixation was achieved using direct Gilmer's wiring and the fractured segments were secured using an eight-hole titanium plate through the existing extraoral wound (Figure 4).

Simultaneously, the patient was put to 30 dives of HBO therapy in a single occupancy chamber, which was pressurized at 2.0 ATA with 100% oxygen three to five days per week over a period of two weeks, following which the patient showed significant improvement. This resulted in the resolution of pain and complete mucosal coverage of the defect (Figure 4).

**Figure 5:**
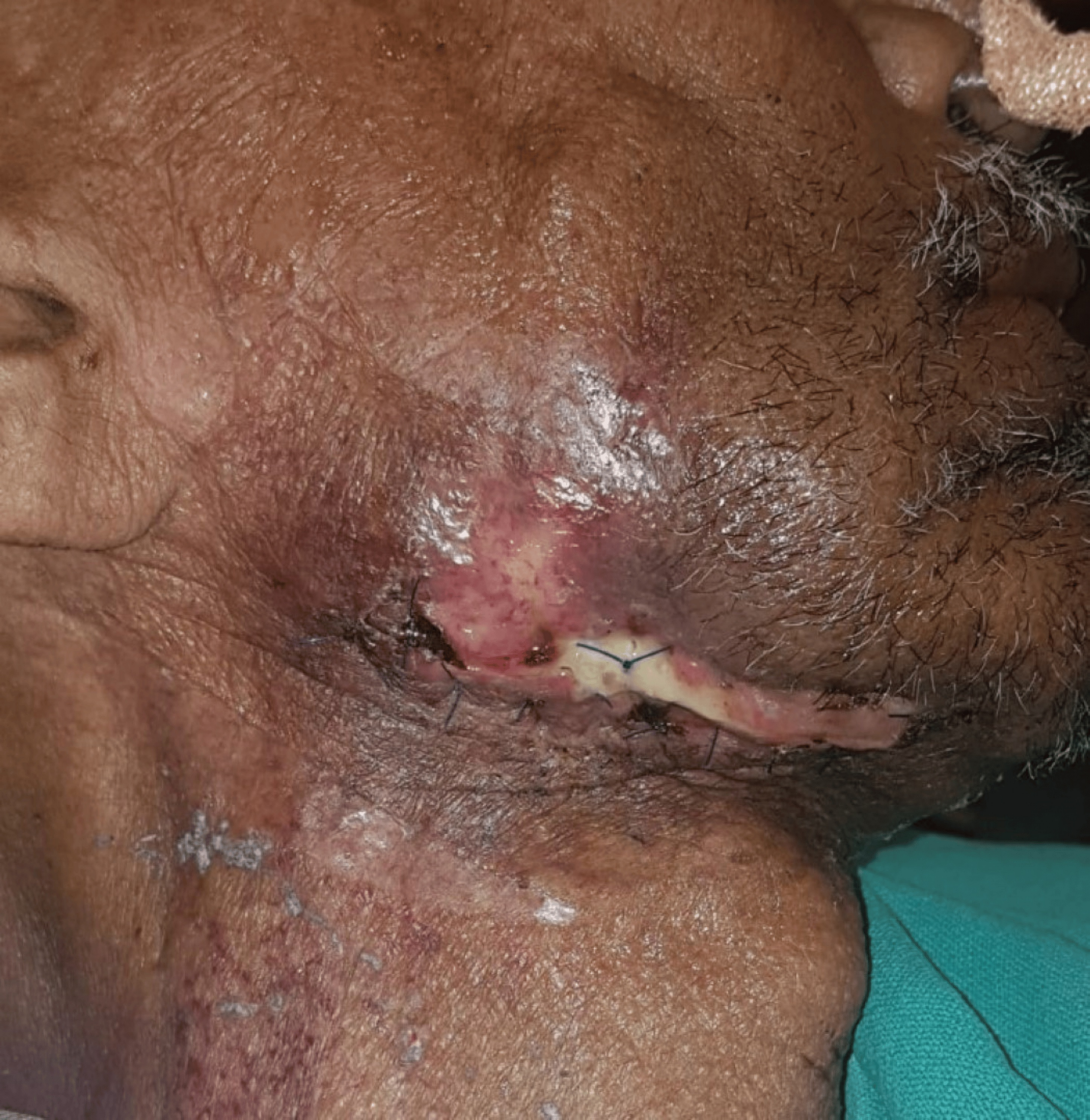
Postoperative wound healing response to hyberbaric oxygen therapy.

The patient was followed up for one and a half years postoperatively, after which he stopped responding to communication.

## Discussion

HBOT has been widely used for many years in treating various medical conditions, owing to its effectiveness in improving oxygen delivery and promoting tissue healing. It is recommended for treating osteoradionecrosis, where bone damage occurs following radiation therapy, and is also effective for managing necrotizing soft tissue infections like gas gangrene. HBOT plays a critical role in supporting compromised skin grafts and flaps by improving their blood supply and survival. Overall, HBOT leverages increased oxygen availability to accelerate healing and address various challenges [4]. The earliest documented case of MRONJ appeared in the early 2000s when Marx and colleagues reported a series of cases involving jawbone necrosis in patients treated with bisphosphonates. This seminal report linked bisphosphonate therapy with osteonecrosis of the jaw, particularly among individuals undergoing treatment for multiple myeloma or breast cancer. This initial finding paved the way for further research and the development of diagnostic criteria and management strategies for MRONJ [5]. These drugs are used for osteoporosis, Paget’s disease, multiple myeloma, and various other metabolic diseases and metastatic neoplasms affecting the bones. The number of patients taking bisphosphonates is on the rise; the dental surgeon thus should be versed in the side effects occurring especially in the oral cavity.

The American Association of Oral and Maxillofacial Surgeons has proposed three key criteria for diagnosis of MRONJ: a) a history of bisphosphonate use, b) exposed and necrotic bone in the jaws lasting at least eight weeks, and c) no prior radiation therapy to the maxillofacial region. In addition, for a diagnosis of MRONJ, the lesions must not exhibit any signs of healing within eight weeks of being detected by a healthcare provider. There are two clinical variants of MRONJ that differ in prevalence, severity, prediction of risk, and treatment: intravenous and oral. The difference in the severity of clinical MRONJ is not due to potency but rather to the route of administration. Drug holiday does not help in the case of intravenous dosage as the drugs reach toxic levels after the third or fourth dose and half-life reaches up to 11 years, whereas oral bisphosphonates are poorly absorbed [2]. The toxic effects of nitrogen-containing bisphosphonates on the normal osteoclast and its precursor cells in the bone marrow result in MRONJ [6]. Bisphosphonates act by exerting their inhibitory effect on osteoclasts, thereby resulting in anti-resorptive action. However, normal bone resorption is pivotal to bone viability. Bone resorption releases specific cytokines that prompt mesenchymal stem cells to differentiate into active bone-forming osteoblasts. These osteocytes, being terminal cells, eventually lose their functional capacity, leading to microfractures in the aging mineral matrix.

The involvement of the jawbone in MRONJ is particularly notable compared to other bones due to three key factors: a) the jawbone is unique in being exposed to the external environment through the periodontal ligament of the teeth; b) the alveolar bone of the jaws remodels at a rate 10 times faster than long bones due to factors such as occlusion and denture wear, making it significantly more reliant on osteoclast-mediated bone remodeling; and c) the thinness of the oral mucosa facilitates breaches in the surface, which can exacerbate the spread of disease and further expose the bone. MRONJ shows a gender predilection for females and is found more frequently in the mandible compared to the maxilla, with a prevalence approximately twice as high in the mandible [7]. MRONJ can present as odontalgia or tooth pain not linked to an odontogenic cause. Patients frequently report a dull, aching pain in the jaw that may radiate to the temporomandibular joint area. In addition, sinus pain may be present, occasionally accompanied by inflammation and thickening of the maxillary sinus wall. Altered neurosensory function, such as numbness or tingling in the oral and jaw areas, is also a notable symptom. In advanced stages of MRONJ, the condition is marked by exposed, necrotic bone or fistulae that extend to the bone, often accompanied by signs of infection. Additional features may include necrotic bone exposure beyond the alveolar region (such as the inferior border and ramus in the mandible or the maxillary sinus and zygoma in the maxilla), pathological fractures, extraoral fistulae, oral-antral or oral-nasal communications, and osteolysis reaching the inferior border of the mandible or the floor of the sinus [8,9,10].

When diagnosing MRONJ, it is important to distinguish it from other conditions that can impact the jaw, such as alveolar osteitis, sinusitis, gingivitis or periodontitis, dental caries, periapical pathology, tooth pain, atypical neuralgias, fibro-osseous lesions, sarcoma, chronic sclerosing osteomyelitis, and temporomandibular joint (TMJ) disorders. In addition, delayed healing and sequestra (i.e., osteonecrosis) can also occur in patients who have not been exposed to antiresorptive agents, underscoring the importance of a thorough evaluation to ensure an accurate diagnosis [11,12]. Most staging systems rely on clinical observations. In 2006, Ruggiero et al. introduced a clinical staging system with three distinct levels, categorized based on the patient's signs and symptoms. This was modified in 2009 and continued through 2014 and 2022 when the AAOMS modified it adding stage 0 to it [2,13]. Radiological alterations are not significant until involving the bone, which is not shown in Stage 0. Thus, proper case history and clinical examination are paramount for the diagnosis of MRONJ. The utility of a preventative drug holiday during tooth extraction for patients on bisphosphonates remains a topic of debate. The management strategies for MRONJ encompass a variety of treatments, including local oral care, pentoxifylline and tocopherol administration, ozone therapy, low-level laser therapy, platelet-rich plasma, parathyroid hormone, bone morphogenetic proteins, antibiotic therapy, and hyperbaric oxygen therapy (HBOT), as well as debridement, resection, reconstructive surgery, and grafting techniques [14,15]. Bone modifiers are also available such as denusumab, teriparatide, raloxifene, and strontium ranalate. HBO therapy enhances wound healing by mobilizing stem cells, reducing edema and inflammation, and accelerating tissue repair. It achieves this by increasing oxygen gradients around ischemic wound areas. The physiological benefits of HBO go beyond simply raising oxygen levels in tissues. Under normal conditions, breathing ambient air produces an arterial oxygen tension of 100 mmHg and a tissue oxygen tension of 55 mmHg. However, inhaling 100% oxygen at 3 ATA can elevate arterial oxygen tension to 2000 mmHg and tissue oxygen tension to 500 mmHg. This substantial increase in oxygen levels enhances the diffusion gradient between tissues and cells, significantly raising cellular oxygen levels. As a result, tissue angiogenesis improves, which, in turn, promotes the development of the collagen matrix [16]. HBOT also improves the oxygen-dependent transport of antibiotics across bacterial cell membranes, interferes with bacterial DNA, and impairs their metabolic processes, making it particularly effective in combating anaerobic bacteria.

In our patient, following HBOT, necrotic tissue and debris healed, the tissue defect was covered, and clinical symptoms such as pain and burning sensation were significantly reduced, with complete resolution of paresthesia [17]. It is essential to evaluate the risk-benefit ratio of HBOT, as studies often understate this. While most side effects are mild and reversible, they can, on rare occasions, be severe and life-threatening. Most side effects of HBOT are self-limiting and can often be prevented with proper screening. The most common pressure-related issue is middle ear barotrauma, which is usually mild and can be avoided through ear-clearing techniques and controlled compression rates. Pulmonary barotrauma is rare and can be prevented with adequate pretreatment evaluation. Oxygen toxicity, although rare, typically presents as a CNS seizure, which resolves with oxygen withdrawal and has no lasting effects. Adjustments to the treatment protocol, such as lowering pressure or adding air breaks, may be considered but are usually unnecessary. Pulmonary oxygen toxicity is not a concern with typical treatments for wound healing. Ocular side effects, such as hyperoxic myopia, are reversible, and patients should wait at least eight weeks post-treatment before getting new prescriptions. Claustrophobia too can be managed with support and, if needed, anxiolytics. Despite these possible risks, HBOT is considered one of the safest approaches for the management of recalcitrant chronic wounds [18,19]. Debridement is regarded as a conservative treatment method, as it focuses on removing only the visibly necrotic tissue while maintaining the integrity of healthy tissue. Its primary goal is to minimize microbial colonization within the wound bed. Antibiotics should be prescribed immediately once the decision to perform debridement is made. Amoxicillin is the drug of choice, but clindamycin or doxycycline could offer the extra benefit of bone penetration. The decision to proceed with sequestrectomy typically depends on the clinical presentation, including the size of the necrotic area, the presence of infection, and the patient's overall response to initial treatment. In advanced MRONJ cases, combining sequestrectomy with debridement can help remove dead bone, reduce infection risk, and promote healing, although it may also require more extensive recovery and rehabilitation [20].

## Conclusions

Patients who are to be put on bisphosphonates should be referred to a dental surgeon in order to address potential problems before starting bisphosphonate treatment. Hyperbaric oxygen provided statistically significant results in our cases, and thus we propose it can be taken up as a primary modality of treatment along with the administration of antibiotics and cessation of BPs. However, more studies with heterogeneous MRONJ stages and larger samples are needed to undergo randomized clinical trials for proper assessment of this therapy and its role in the treatment of MRONJ.
